# Context-sensitive fluid therapy in critical illness

**DOI:** 10.1186/s40560-016-0150-7

**Published:** 2016-03-15

**Authors:** Tsuneo Tatara

**Affiliations:** Department of Anesthesiology and Pain Medicine, Hyogo College of Medicine, 1-1 Mukogawa-cho, Nishinomiya, Hyogo 663-8501 Japan

**Keywords:** Microcirculation, Glycocalyx, Fluid, Hydrostatic pressure, Surgery, Trauma, Sepsis

## Abstract

Microcirculatory alterations are frequently observed in critically ill patients undergoing major surgery and those who suffer from trauma or sepsis. Despite the need for adequate fluid administration to restore microcirculation, there is no consensus regarding optimal fluid therapy for these patients. The recent recognition of the importance of the endothelial glycocalyx layer in capillary fluid and solute exchange has largely changed our views on fluid therapy in critical illness. Given that disease status largely differs among critically ill patients, fluid therapy must not be considered generally, but rather tailored to the clinical condition of each patient. This review outlines the current understanding of context-sensitive volume expansion by fluid solutions and considers its clinical implications for critically ill patients. The modulation of capillary hydrostatic pressure through the appropriate use of vasopressors may increase the effectiveness of fluid infusion and thereby reduce detrimental effects resulting from excessive fluid administration.

## Introduction

In critically ill patients, including high-risk patients undergoing major surgery and patients with trauma or sepsis, adequate fluid administration is essential for the maintenance of tissue perfusion. Nevertheless, there is no consensus regarding the optimal fluid therapy (e.g., fluid type, volume, and timing of administration) for these patients [1]. Hydroxyethyl starch (HES) solutions are widely used to restore intravascular volume in high-risk patients undergoing anesthesia for major surgery, but the use of HES during perioperative period is controversial because of potential adverse events including renal dysfunction [2]. In traumatic patients, the question remains whether limiting volumes of early resuscitation with permissive hypotension improves outcomes from trauma [3]. The benefit of early goal-directed bolus fluid therapy in septic patients has been reappraised in recent treatment bundles for septic care [3].

Key features of fluid therapy in critical illness are vascular content (i.e., intravascular volume), vascular tone (i.e., vasoconstriction or vasodilation), and capillary permeability determined by endothelial integrity [4]. Surgical insults and severe infections affect all of these features, the magnitudes of which depend on clinical context such as the phase of illness. For example, fluid administration increases mean systemic filling pressure through intravascular volume expansion, thus increasing venous return and cardiac output [5, 6]. However, the degree of intravascular volume expansion is context-sensitive as demonstrated by larger plasma volume expansion following fluid infusion in the hypovolemic state compared to the normovolemic state [7].

Critically ill patients show a wide variety of pathophysiological conditions, severity of disease, and phase of progress. Thus, consideration of the context-sensitive volume effects of fluids may shed light on the long-standing controversy surrounding fluid therapy in critically ill patients.

## Review

### Physiological basis

#### Endothelial glycocalyx and endothelial surface layer

The endothelium is covered with a gel-like layer of endothelial glycocalyx (EG), which is a luminal coat of biopolymers forming a negatively charged meshwork [8–10]. The EG layer consists of a variety of endothelial membrane-bound molecules, including glycoproteins and proteoglycans carrying negatively charged glycosaminoglycans (i.e., heparan sulfate, chondroitin sulfate, and hyaluronic acid). The EG layer plays an important role in fluid and solute movement across capillaries, mechanotransduction that couples shear stress to endothelial cell responses, and neutrophil adhesion to the endothelial cell surface [11]. The EG structure is fairly stable under physiological conditions, striking a balance between the synthesis of new glycans and shear-dependent shedding of exiting glycans. Degradation of the EG layer is closely associated with the pathophysiology of inflammation, capillary leakage, and edema formation in surgical injuries and disease states, including ischemia-reperfusion injury, sepsis, trauma, and hypervolemia [11]. Once destroyed, full restitution of the EG layer requires several days [12].

The EG layer is in a dynamic equilibrium with plasma proteins, forming a flexible gel-like structure called the endothelial surface layer (ESL) [8]. The ESL acts as a barrier to fluid and large molecules, has a thickness even achieving a magnitude of over 1 μm, and thus physiologically occupies approximately 25 % of the total intravascular space [12]. Consistent with this, Vink and Duling [13] demonstrated that dextrans with a molecular weight larger than 70,000 Da failed to penetrate the ESL in hamster cremaster muscle capillaries (ESL thickness 0.4–0.5 μm), whereas dextrans with a molecular weight of 40,000 Da equilibrated with the ESL within 1 min, evidencing the important role of the ESL as a molecular filter.

#### Capillary hydrostatic pressure

**Fig. 1:**
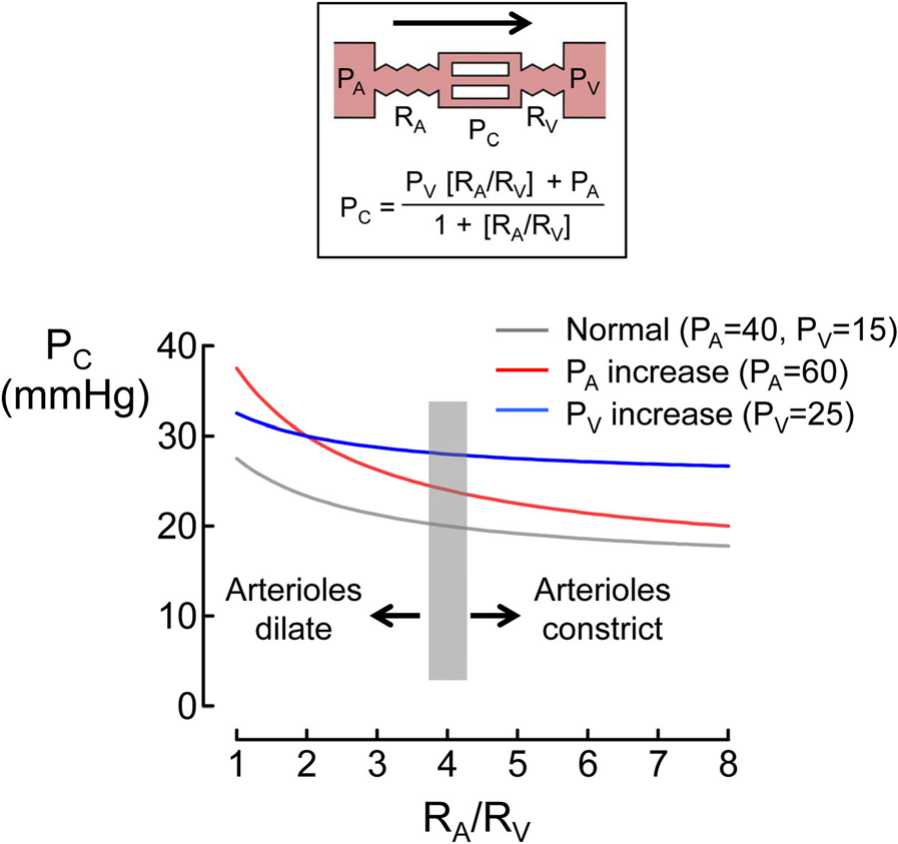
Theoretical prediction of capillary hydrostatic pressure as a function of ratio of hydraulic resistance in arterioles to venules. Capillary hydrostatic pressure (*P*_C_) was obtained from the equation in the *box* on *P*_C_ (i.e., the Pappenheimer-Soto Rivera equation). The equation is determined by hydrostatic pressures in the arteriole (*P*_A_) and venule (*P*_V_) and the ratio of hydraulic resistance of the arteriole (*R*_A_) to that of the venule (*R*_V_) (i.e., *R*_A_/*R*_V_) assuming that blood flow is constant through the capillary (*arrow* in the figure in the box). Increased *P*_A_ (without change of *P*_V_) or increased *P*_V_ (without change of *P*_A_) increases *P*_C_ compared to the normal state. Vasodilation increases *P*_C_ (i.e., leftward in the *P*_C_ curve), while vasoconstriction decreases *P*_C_ (i.e., rightward in the *P*_C_ curve). *P*_C_ varies in a rather complicated fashion in the clinical setting due to different changing patterns of *P*_A_, *P*_V_, and *R*_A_/*R*_V_. The pressure values of *P*_A_ and *P*_V_ are expressed in mmHg. *Shaded area* denotes the normal value of *R*_A_/*R*_V_

Due to capillary hydraulic resistance, blood pressure falls along a capillary from the arterial end (e.g., 32–36 mmHg for human skin) to the venous end (e.g., 12–25 mmHg for human skin), and thus, capillary hydrostatic pressure (*P*_C_) lies between hydrostatic pressure in the arteriole (*P*_A_) and that in the venule (*P*_V_) [14]. As blood flow from arterioles to mid-capillaries (i.e., [*P*_A_–*P*_C_]/*R*_A_, where *R*_A_ is hydraulic resistance in arterioles) is equal to the blood flow from mid-capillaries to venules (i.e., [*P*_C_–*P*_V_]/*R*_V_, where *R*_V_ is hydraulic resistance in the venule) in the steady state, *P*_C_ can be described by an equation (Pappenheimer-Soto Rivera) that includes *P*_A_, *P*_V_, and the ratio of precapillary arteriolar resistance to post-capillary venular resistance [*R*_A_/*R*_V_] (see box in Fig. 1) [14]. The increase of *P*_A_ or *P*_V_ increases *P*_C_, but as *R*_A_/*R*_V_ is large (~4) under normal conditions, *P*_C_ is more sensitive to *P*_V_ than *P*_A_ and is more similar to *P*_V_ than *P*_A_ (Fig. 1). Further increase in *R*_A_/*R*_V_ due to vasoconstriction drops *P*_C_ (i.e., rightward in the *P*_C_ curve in Fig. 1), whereas a decrease in *R*_A_/*R*_V_ due to vasodilation increases *P*_C_ (i.e., leftward in the *P*_C_ curve in Fig. 1) [14]. Given that mean arterial pressure (MAP) and intravascular volume modulate *P*_A_ and *P*_V_, and a balance of vascular tones in the arteriole and venule determines *R*_A_/*R*_V_, *P*_C_ varies in a rather complicated fashion in the clinical context [15]. For example, sodium nitroprusside and nitroglycerin, both of which are vasodilators, affect *P*_C_ differently [16]. When MAP was reduced to 40 mmHg by these drugs in striated muscle vessels in hamsters, sodium nitroprusside increased *P*_C_ from 22 mmHg (i.e., baseline) to 26 mmHg, whereas nitroglycerin decreased *P*_C_ from 22 mmHg (i.e., baseline) to 17 mmHg. Given that both drugs decreased *R*_A_ by 80 % compared to the baseline, the difference was explained by the contrasting effects of these drugs on *R*_V_ in that sodium nitroprusside increased *R*_V_ by 40 % whereas nitroglycerin decreased *R*_V_ by 40 % compared to the baseline.

#### Effects of capillary hydrostatic pressure on capillary fluid filtration and colloid permeation

According to the revised Starling equation, transendothelial pressure differences and plasma-subglycocalyx colloid osmotic pressure (COP) differences are central to fluid filtration, with interstitial COP being negligible [14, 17].

**Fig. 2:**
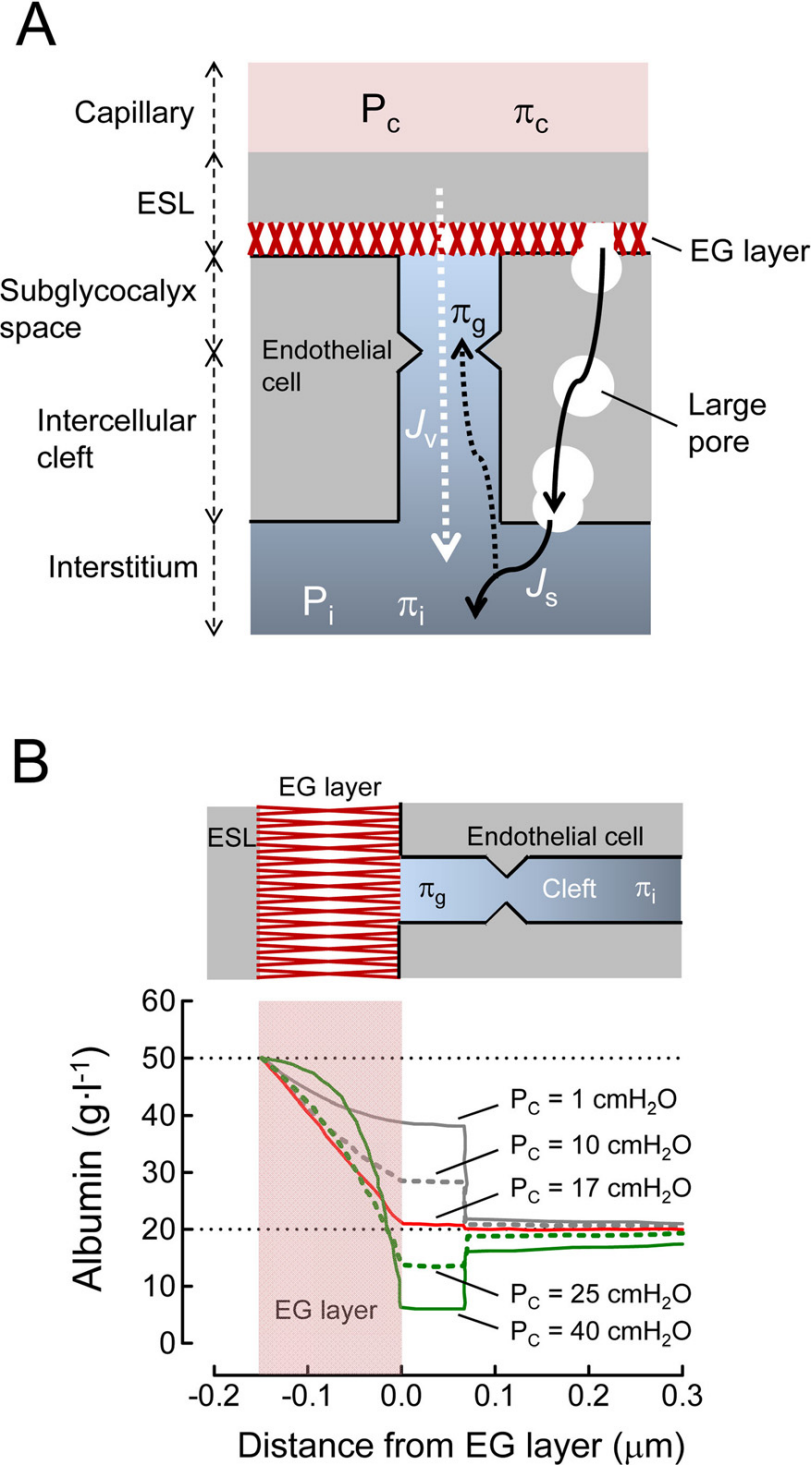
**a** Effects of fluid filtration (*J*_v_) across capillaries on subglycocalyx albumin concentration. Albumin entering into the interstitium via a large pore (*J*_s_, *solid arrow in black*) diffuses backwards into the subglycocalyx space according to concentration gradients (i.e., upstream diffusion, *dotted arrow in black*). Fluid filtration across the endothelial glycocalyx (EG) layer dilutes albumin in the subglycocalyx space (i.e., downstream washout, *dotted arrow in white*). Cited from reference [17] with permission. *ESL* endothelial surface layer, *P*_*C*_, *P*_*i*_ hydrostatic pressure in the capillary and interstitium, respectively, *π*_*c,*_ *π*_*i,*_ *π*_*g*_ colloid osmotic pressure in the capillary, interstitium, and subglycocalyx space, respectively. **b** Effects of capillary hydrostatic pressure (*P*_C_) on albumin concentration along the endothelial glycocalyx (EG) layer. *ESL* endothelial surface layer, *π*_*i,*_ *π*_*g*_ colloid osmotic pressure in the interstitium and subglycocalyx space, respectively. Normal *P*_C_ is approximately 25 cmH_2_O. Cited from reference [18] with permission

Proteins in plasma can diffuse into the interstitium via large pores (50–60 nm in diameter) in fenestrated capillaries (e.g., capillaries in kidneys and intestinal mucosa). Fenestrated capillaries are at least an order of magnitude more permeable to water and small hydrophilic solutes than continuous capillaries (e.g., capillaries in skeletal muscle, skin, and lungs) [14]. Subglycocalyx protein concentration that determines COP in the subglycocalyx space is determined by the rates of upstream diffusion and downstream washout [14, 17] (Fig. 2a). Under a normal *P*_C_ (approximately 25 cmH_2_O) and filtration rate, COP in the subglycocalyx space may be 70–90 % of that in the interstitium. However, at a low filtration rate, plasma proteins entering the interstitium via the large pore accumulate there, raising the interstitial protein concentration. Accumulated plasma protein diffuses more easily up the cleft, raising the concentration of subglycocalyx protein (i.e., upstream diffusion). In contrast, a high filtration rate dilutes subglycocalyx protein via downstream washout, leading to a decrease in subglycocalyx protein concentration (Fig. 2a).

These features in fluid filtration across the EG layer affect fluid movement during fluid administration. At subnormal *P*_C_, transcapillary flow approaches zero with a minimal COP difference [18, 19] (Fig. 2b). In this situation, both crystalloid and colloid solutions are retained in the intravascular space until transcapillary flow resumes [19, 20]. In contrast, at supranormal *P*_C_, the COP difference is maximal, and thus, fluid movement depends on the transendothelial pressure difference (Fig. 2b). When a colloid solution is infused in this situation, it maintains COP by distributing through the plasma while increasing *P*_C_, and this increases fluid filtration. A crystalloid solution in the same situation lowers plasma COP but increases *P*_C_, and thus, fluid filtration increases more than with a colloid solution [19, 20].

*P*_C_ can affect capillary permeability of colloid molecules. A mathematical model describing fluid and albumin fluxes in the EG layer demonstrated that in the steady state, a slight reabsorption of albumin (i.e., from the subglycocalyx space to the capillary) occurs at low *P*_C_, whereas albumin convection flux (i.e., from the capillary to the interstitium) increases at high *P*_C_ [18]. Chen and Fu [21] developed an electrodiffusion model describing the transport of macromolecules across the EG layer. The model demonstrated that albumin permeability across the layer is attenuated by the negative charge of EG, and the increase in *P*_C_ from 15 cmH_2_O to 30 cmH_2_O doubles albumin permeability across the EG layer through a convection mechanism. Increases in macromolecule permeability across the EG layer at high *P*_C_ is also observed with neutral macromolecules (e.g., HES) [21]. The larger the macromolecule, the larger the effect *P*_C_ has on permeability across the EG layer. These findings suggest that *P*_C_ plays a crucial role in volume expansion by colloid solutions via the control of fluid filtration and permeation of colloid molecules across the EG layer.

**Fig. 3:**
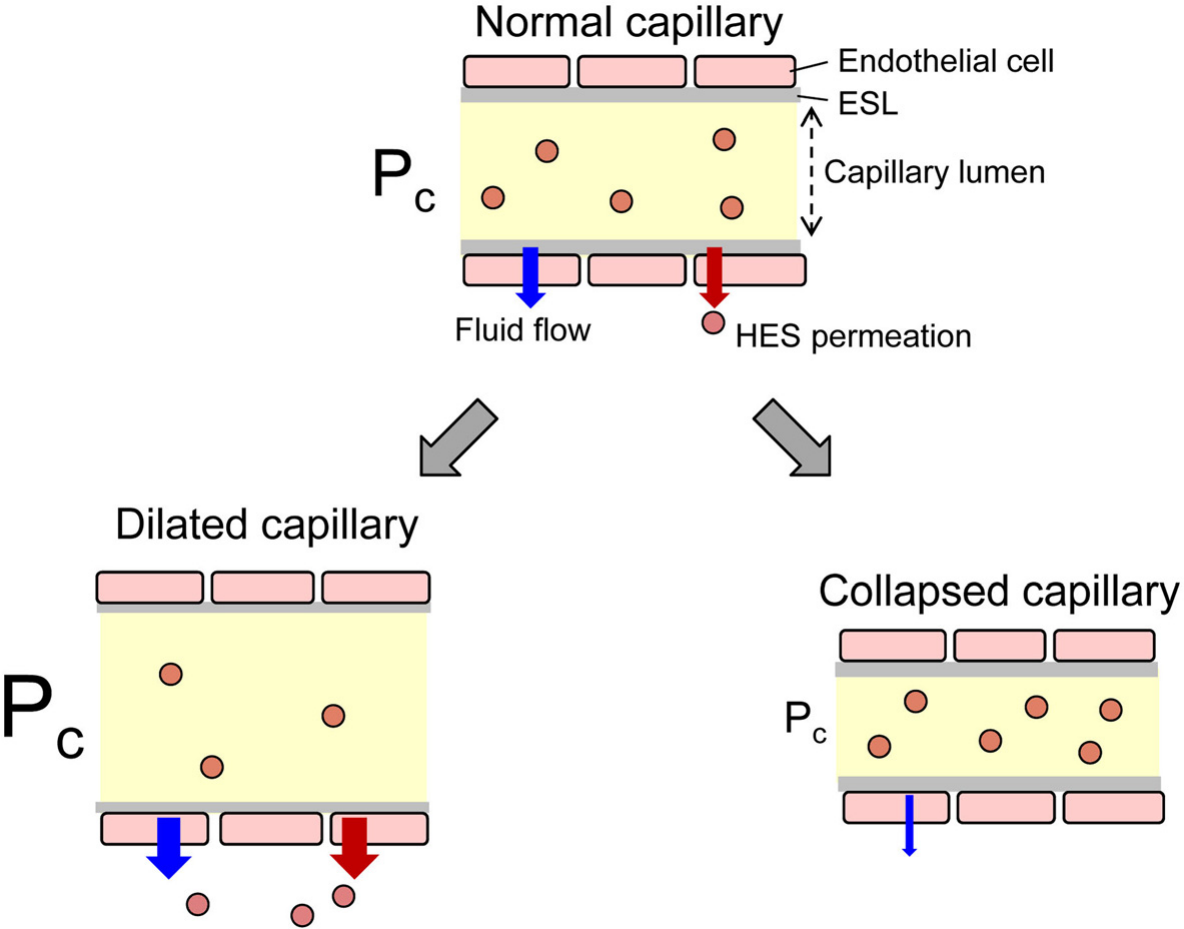
Proposed effects of capillary hydrostatic pressure (*P*_C_) on fluid flow and hydroxyethyl starch (HES) permeation across capillaries. The increase in *P*_C_ resulting from vasodilation increases fluid flow and HES permeation across capillaries (*lower left panel*). The use of vasopressors attenuates the increases in fluid flow and HES permeation across capillaries by normalizing *P*_C_. When *P*_C_ is low in collapsed capillary under hypovolemia, most of the infused HES solution is retained in the intravascular space (*lower right panel*), whereas vasopressor worsens tissue perfusion by further lowering *P*_C_. *ESL* endothelial surface layer

These characters might provide us a new insight to a choice of colloid solution infusion or vasopressor use for critically ill patients. When *P*_C_ is high in well-perfused dilated capillaries in the conditions such as after fluid volume loading at the induction of anesthesia and early sepsis after fluid volume loading, volume effect of colloid solution is reduced (Fig. 3, lower left panel). In this context, lowering *P*_C_ to the normal value by appropriate use of vasopressor can increase volume effect of colloid solution. Contrarily, *P*_C_ is low in collapsed capillary in the hypovolemic conditions such as hemorrhage shock during surgery or trauma and early sepsis before fluid volume loading. Most of the infused colloid solution is retained in the intravascular space, whereas excessive use of vasopressor can worsen tissue perfusion by further lowering *P*_C_ (Fig. 3, lower right panel).

### Experimental and clinical evidence

#### Context-sensitive volume effects of fluid solutions

It was long believed that only 20 % of infused crystalloid remains in the intravascular space, whereas most colloid solutions, such as HES solution, remain in the intravascular space. Although this is true for healthy individuals, it is not the case for patients with hemorrhage or those undergoing general anesthesia.

**Fig. 4:**
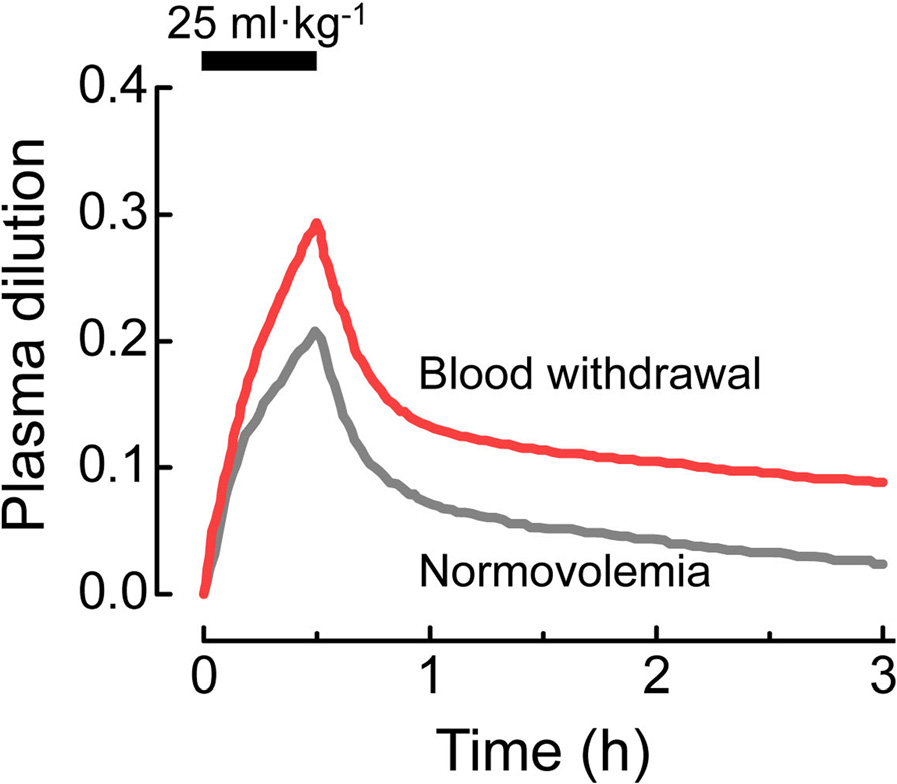
Time course of plasma dilution following crystalloid infusion in volunteers under normovolemia and after blood withdrawal. Ringer’s acetate solution (25 ml kg^−1^) was intravenously infused over 30 min to male volunteers when they were normovolemic and after 900 ml of blood had been withdrawn. Plasma dilution (no unit) was indicated by blood hemoglobin changes. Cited from reference [23] with permission

Given that plasma volume measurement using the dye dilution technique is time-consuming and thus not suitable for real-time measurement, a volume kinetics method based on changes in hemoglobin concentration has been developed to measure plasma volume changes after fluid administration [22]. In males, plasma dilution following administration of 25 ml kg^−1^ Ringer’s acetate solution, as assessed by changes in hemoglobin concentration, was larger after 900 ml of blood had been withdrawn compared to when they were normovolemic [23] (Fig. 4). Volume kinetic analysis revealed that the observed increase of plasma volume expansion for crystalloid solution in the hypovolemic state (i.e., after blood withdrawal) can be attributed to a decrease in elimination clearance from the intravascular space. The increase in retention of crystalloid solution in the intravascular space was also observed during general [24, 25], spinal [24], and epidural [25] anesthesia. This effect was attributed to a decrease in fluid shift from the intravascular space to the extravascular space (i.e., interstitium), the magnitude of which increased with the decrease of MAP [22].

The context-sensitive volume effect also applies to colloid solutions.

Rehm et al. [26] measured blood volume changes during acute fluid volume loading of 6 % HES 200/0.5 (average molecular weight, 200,000 Da) and 5 % albumin solutions (20 ml kg^−1^ over 15 min) at the induction of general anesthesia. Plasma volumes were measured before and 30 min after the end of fluid infusion by the dilution technique using indocyanine green and hematocrit changes. Increases of blood volume after volume loading of HES 200/0.5 and 5 % albumin solutions were 43 and 38 % of the infused fluid volume, respectively. These volume effects were much smaller compared to those during isovolemic hemodilution (20 ml kg^−1^ of blood withdrawal) in that approximately 90 % of infused HES 200/0.5 and albumin solutions were retained in the intravascular space [7, 27].

**Fig. 5:**
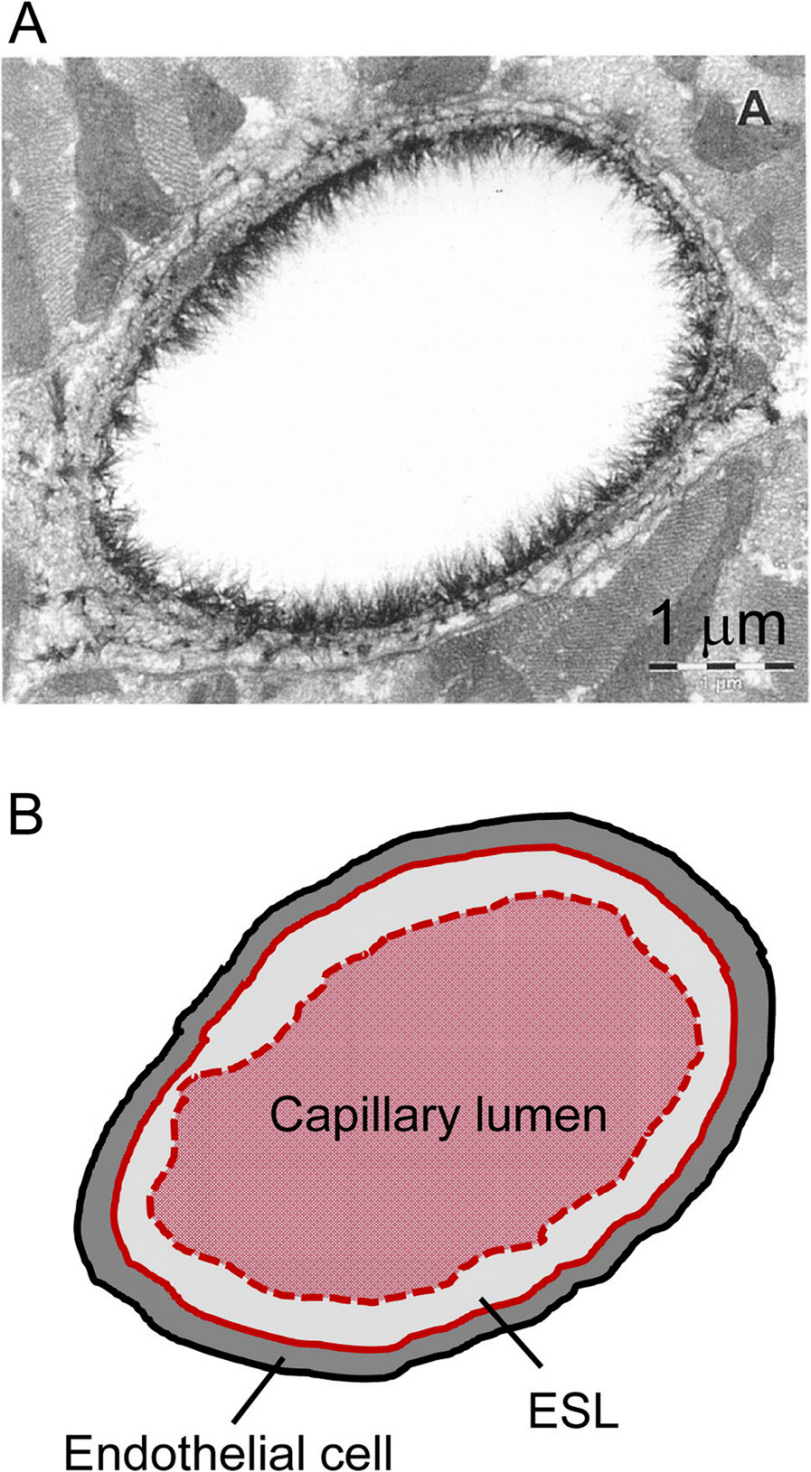
**a** Electron micrograph showing the endothelial glycocalyx in a capillary of the rat left ventricular myocardium, as indicated by an Alcian blue 8GX staining. Cited from reference [9] with permission. **b** Schema representing a difference of plasma volume measured by dye dilution and hematocrit dilution. Dye (i.e., indocyanine green) diffuses into the endothelial surface layer (ESL) and thus measures plasma volume inside the *red thick line*, whereas hematocrit reflects plasma volume excluding the ESL (*red dotted line*)

The most remarkable finding in that study [26] was that the ratio between whole body hematocrit (i.e., erythrocyte volume divided by the sum of plasma volume and erythrocyte volume) and large vessel hematocrit (i.e., hematocrit of arterial blood sample) significantly increased after fluid volume loading at the induction of general anesthesia compared to before infusion (0.95 vs. 0.84 for HES 200/0.5; 0.93 vs. 0.83 for albumin). This ratio reflects that noted between distribution spaces for indocyanine green and erythrocytes, in which indocyanine green distributes into the ESL due to its low molecular weight, whereas erythrocytes cannot distribute into this layer (Fig. 5). Accordingly, the increase in ratio between whole body hematocrit and large vessel hematocrit after colloid infusion suggests that volume loading of colloid solutions thinned the ESL. The authors proposed that these effects of colloid solutions on the ESL were caused by a release of atrial natriuretic peptide during iatrogenic acute hypervolemia. This scenario was confirmed by another clinical study showing that volume loading of 6 % HES 130/0.4 solutions (20 ml∙kg^−1^ over 15 min) increased release of atrial natriuretic peptide (by 100 %) and increased serum concentrations of hyaluronic acid and syndecan-1 (both by 80 %), both of which are constituents of the ESL [28]. Consistent with this, pig studies have found that atrial natriuretic peptide induces the shedding of the EG layer and enhances vascular permeability [29, 30].

Clinically, perturbations of the EG layer and ESL can range from deterioration of the ESL (e.g., aggressive fluid administration) to destruction of the EG layer (e.g., sepsis) [12]. Septic shock patients exhibited an increase in plasma levels of heparan sulfate and hyaluronic acid to fourfold that of controls, whereas levels of chondroitin sulfate did not differ between groups [31]. The enzymatic removal of glycosaminoglycans of the EG layer in post-capillary venules in rats reduced the thickness of the EG layer, but the effects on solute permeability of the EG layer were inconsistent across enzymes [32]. Chondroitinase and hyaluronidase increased the permeability of dextran with a molecular weight of 70,000 Da across the EG layer, whereas heparinase decreased it. This discrepancy may be explained by a collapse of the EG layer due to the removal of heparan sulfate, which compacts the EG layer to maintain a constant resistance to filtration [32].

#### Effects of fluid rate on plasma volume expansion

Under conditions that cause capillary leakage, such as sepsis, the infusion rate of colloid solution influences the volume expansion effects of colloid solutions. Rapid infusion of colloid solutions can transiently increase arterial and venous pressures. The resultant increase in *P*_C_ augments capillary leakage of fluid and colloids from the intravascular space to the interstitium, and thus reduces the volume expansion effects of colloid solutions. This hypothesis was supported for albumin, gelatin, and HES 130/0.4 (average molecular weight 130,000 Da) in a septic rat model [33], and for dextran and albumin in a septic pig model [34]. In the former study, rapid infusion of 5 % albumin solution and 6 % HES 130/0.4 solution (12 ml kg^−1^ over 15 min) increased MAP but decreased plasma volume expansion at 3 h after the initiation of fluid infusion compared to slow infusion of these solutions (12 ml kg^−1^ over 3 h) (−3 vs. 3 ml kg^−1^ for albumin; −6 vs. −2 ml kg^−1^ for HES 130/0.4).

#### Effects of vasopressors on plasma volume expansion

The dependence of plasma volume on MAP was demonstrated in post-cardiac surgery patients who required norepinephrine to treat vasodilatory shock [35]. The infusion rates of norepinephrine were randomly adjusted to maintain MAP at 60, 75, or 90 mmHg for 30 min. Plasma volume calculated by hematocrit changes decreased by 6.5 and 9.4 % when MAP was increased from 60 to 75 mmHg and from 60 to 90 mmHg, respectively. Norepinephrine, in general, decreases *P*_C_ via arteriole vasoconstriction (Fig. 1) but can also constrict venules. The resultant increase in *P*_V_ may increase *P*_C_ under conditions of increased MAP (i.e., *P*_A_). Therefore, these findings suggest that the increase in *P*_C_ resulting from a norepinephrine-mediated increase in MAP augments transcapillary fluid extravasation, leading to plasma volume loss.

These effects of norepinephrine on plasma volume changes depend on intravascular volume. In one study using rats with increased capillary permeability due to anaphylactic reaction, plasma volume changes following the infusion of 5 % albumin solution were measured with the albumin tracer technique [36]. The norepinephrine-induced increase in blood pressure reduced plasma volume, the magnitude of which was much greater under increased capillary permeability compared to normal capillary permeability. However, the plasma-reducing effect of norepinephrine was less pronounced under hypovolemia, suggesting that the decrease in *P*_C_ due to hypovolemia results in the retention of more fluid in the intravascular space, thereby attenuating the plasma-reducing effect of norepinephrine compared to the normovolemic state.

### Context-sensitive fluid therapy

#### Gap between macro-hemodynamics and microcirculation

The restoration of microcirculation is essential for the improvement of outcomes in critically ill patients. Nevertheless, as the assessment of microcirculation at the bedside is difficult, more readily measureable macro-hemodynamic parameters, such as arterial pressure and cardiac output, are used as surrogates, with the assumption that microcirculatory perfusion is coupled to macro-hemodynamics. However, in shock states arising from sepsis and hemorrhage, the relationship is disrupted such that microcirculatory organ perfusion may be abnormal despite restoration of seemingly adequate macro-hemodynamic parameters [37].

In traumatic hemorrhage shock patients, despite restoration of macro-hemodynamics, sublingual microcirculation was impaired for at least 72 h [38]. Given that this applies to major surgery with massive hemorrhage, the restoration of macro-hemodynamic circulation may not equate to the preservation of microcirculation. In patients undergoing high-risk major abdominal surgery, the density and proportion of sublingual perfused capillaries was lower in patients who subsequently developed postoperative complications than those with an uneventful postoperative course [39].

#### Phase of illness

Critically ill patients largely differ not only by a type of insult but also by disease phase. Hoste et al. [40] proposed four phases of intravenous fluid therapy for critically ill patients: *rescue*, *optimization*, *stabilization*, and *de-escalation*. The “rescue” phase involves aggressive administration of fluid solution for the immediate management of life-threatening conditions associated with impaired tissue perfusion, such as septic shock and major trauma. The “optimization” phase involves adjusting the fluid type, rate, and amount based on clinical condition in order to optimize tissue perfusion, such as during major surgery. The “stabilization” phase aims for a zero or negative fluid balance by minimal maintenance infusion, such as during stays at the intensive care unit (ICU) after major surgery. The “de-escalation” phase involves minimization of fluid administration and mobilization of extra fluids to optimize fluid balance, such as during the recovery phase.

#### Anesthesia

**Fig. 6:**
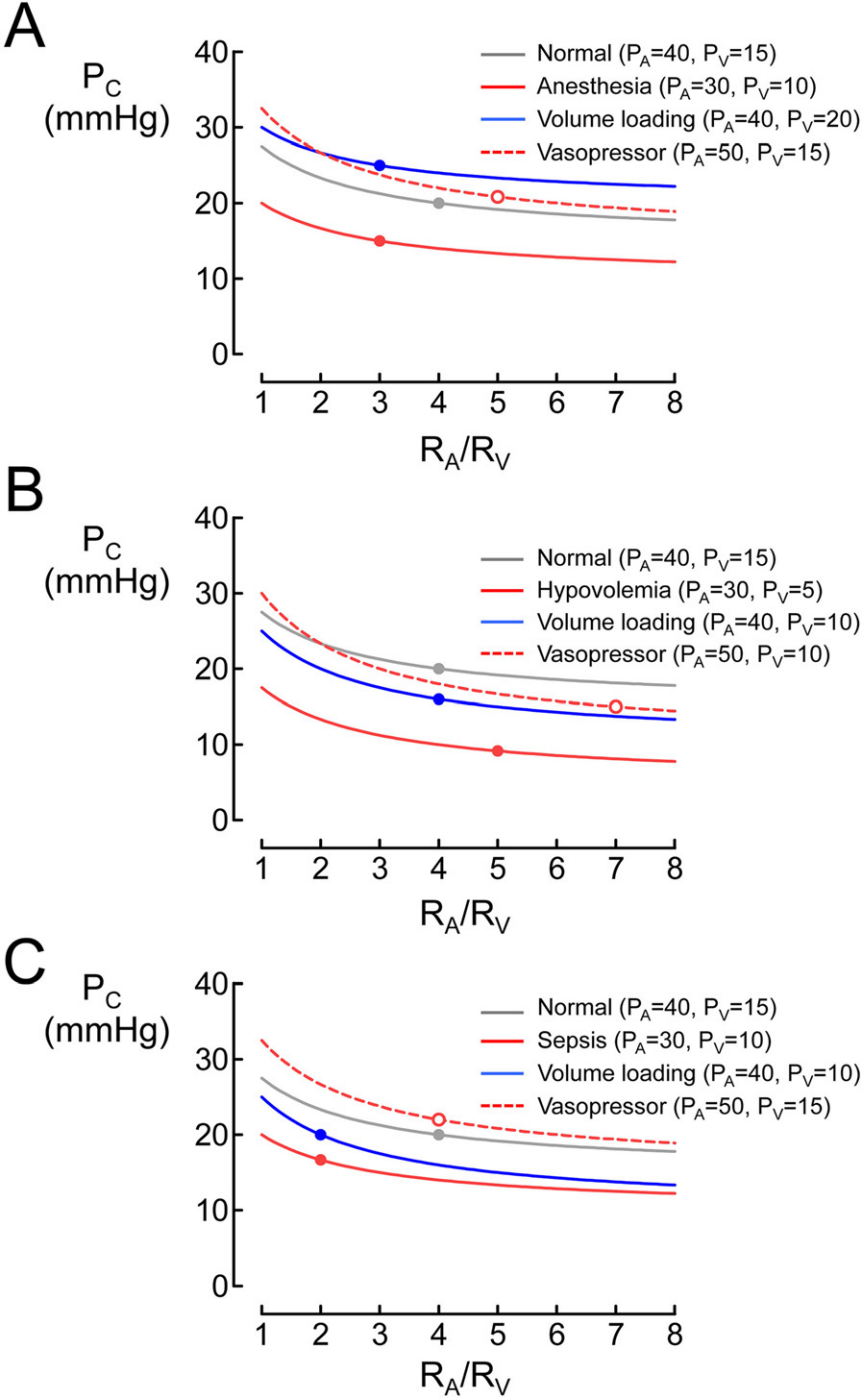
Theoretical prediction of capillary hydrostatic pressure as a function of ratio of hydraulic resistance in arterioles to venules during general anesthesia (**a**), in the hypovolemic state (**b**), and in sepsis (**c**). The values of capillary hydrostatic pressure (*P*_C_) were calculated as a function of the ratio of hydraulic resistance of the arteriole (*R*_A_) to that of the venule (*R*_V_) (i.e., *R*_A_/*R*_V_) using the equation in the *box* (Fig. 1) on *P*_C_. *P*_C_ values were simulated during general anesthesia (**a**), in the hypovolemic state (**b**), and in sepsis (**c**) after intervention of fluid volume loading or use of vasopressor at given hydrostatic pressures (mmHg) in the arteriole (*P*_A_) and venule (*P*_V_). *Circle symbols* in the curves denote assumed values of *R*_A_/*R*_V_

Most general anesthetics have vasodilating action [41, 42]. It has been a common practice to administer a large amount of fluid to treat the resultant hypotension, especially at the induction of general anesthesia. However, this treatment lacks rationale because overnight fasting does not significantly decrease plasma volume in low-risk surgical patients [43]. Given that *P*_C_ is increased by vasodilation and fluid volume loading at the induction of anesthesia (Fig. 6a, blue line), the volume effect of infused fluid is attenuated as a result of increased fluid filtration and colloid permeation across capillaries. Accordingly, the rational therapy for hypotension caused by anesthetics is the appropriate use of vasopressors that normalize the decreased vascular tone (Fig. 6a, red broken line), allowing for the retention of more infused fluid in the intravascular space.

Vasopressors, such as norepinephrine, increase tissue perfusion pressure, but there remains a potential risk that the resulting vasoconstriction impairs microcirculatory blood flow in vulnerable organs, such as the intestinal tract and kidneys, to hypovolemia. In a pig model of abdominal surgery (i.e., laparotomy of 4-h duration) with low volume replacement (3 ml kg^−1^ h^−1^ of Ringer’s lactate solution), the infusion of norepinephrine to increase MAP to 65 mmHg (0.04 μg kg^−1^ min^−1^) and 75 mmHg (0.12 μg kg^−1^ min^−1^) did not adversely affect microcirculatory blood flow or tissue oxygen in the intestinal tract [44]. However, given that hypovolemia was not remarkable in that model, this result cannot be extrapolated to conditions of severe hypovolemia arising from massive hemorrhage during prolonged major abdominal surgery or those requiring higher infusion rates of norepinephrine.

Sufficient cardiac preload is indispensable for optimizing cardiac output.

Cardiac preload is determined by venous return, which is equal to the difference between mean systemic filling pressure and right atrial pressure divided by the resistance to venous flow return [6]. Veins of the systemic circulation contain approximately 70 % of the blood volume in the whole body. This highly distensible fluid component consists of an unstressed volume and a stressed volume, occupying approximately 70 and 30 % of the total volume of the venous system, respectively [5]. The unstressed volume is the volume of blood in a vein at transmural pressure equal to zero (i.e., pressure within the vessel is equal to that outside the vessel) and thus does not directly contribute to venous return. In contrast, the stressed volume represents blood volume above the opening and thus contributes directly to mean systemic filling pressure and venous return. Changes in vascular tone alter the ratio of unstressed to stressed volume [5, 6]. Specifically, venoconstriction shifts blood from unstressed volume to stressed volume, whereas venodilation shifts blood from stressed volume to unstressed volume. Hemorrhage decreases stressed volume, while fluid administration increases it.

Given that general anesthetics decrease venous tone as confirmed for pentobarbital [45] and volatile anesthetics (e.g., sevoflurane) [46], anesthetics increase unstressed volume and decrease stressed volume. Resultant decrease of mean systemic filling pressure decreases venous return and thus cardiac output. In this context, a large amount of fluid volume loading is required to increase mean systemic filling pressure to the level that restores venous return to the normal value. Therefore, venoconstriction by appropriate use of vasopressor is rational because it recruits blood from enlarged unstressed volume to stressed volume and thus increases mean systemic filling pressure and venous return.

#### Surgery

Ongoing hemorrhage and fluid shifts from the intravascular space to the interstitium due to surgical trauma cause hypovolemia during major abdominal surgery. However, a higher infusion rate of crystalloid solution (i.e., >10 ml kg^−1^ h^−1^), which is routine practice for major abdominal surgery, does not contribute to the recovery of plasma volume but enhances interstitial edema [47]. This phenomenon depends on surgical duration. In a mathematical simulation for abdominal surgery, the range of crystalloid infusion rates required to maintain plasma volume and interstitial volume within critical values (>−15 and <20 % of baseline values, respectively) was wide for short-duration surgery (2–19 ml kg^−1^ h^−1^ for a 2-h surgery), whereas it was narrow for long-duration surgery (5–8 ml kg^−1^ h^−1^ for a 6-h surgery) [48].

Hypovolemia resulting from major abdominal surgery decreases *P*_C_ (Fig. 6b, red line). As the plasma volume expanding effect of the infused fluid is increased in this context, fluid administration is the first choice for treating hypotension resulting from hypovolemia (Fig. 6b, blue line). Indeed, vasopressor can restore *P*_C_ and maintain microcirculation by increasing MAP (Fig. 6b, red broken line) and recruiting blood from the unstressed volume to the stressed volume. However, in cases of massive hemorrhage (e.g., >20 ml kg^−1^), unstressed blood volume is already reduced by an increase in sympathetic discharge as compensation to the decreased circulating blood volume. In this context, aggressive use of vasopressor is detrimental given that it does not induce further recruiting of blood from the unstressed volume and thus impairs microcirculation by further lowering *P*_C_ [49].

However, aggressive fluid infusion transiently causes hypervolemia, leading to a decrease in the volume expanding effects of the aforementioned fluid solution [7]. Therefore, infusion of a relatively small volume of fluid solution (e.g., 3–4 ml kg^−1^) over a short time (5–10 min) should be repeated while observing hemodynamic responses to the infusion. Many recent clinical studies have shown that goal-directed fluid therapy according to fluid responsiveness (i.e., increase in stroke volume or cardiac output following fluid bolus infusion above a certain level) enables the stabilization of intraoperative hemodynamics and improvement of postoperative outcomes following major abdominal surgery [50–52]. In fluid therapy, fluid volume loading is typically repeated until fluid responsiveness disappears, assuming that the heart is on the steep portion of the Frank-Starling curve while fluid responsiveness is observed [53, 54]. However, the application of this strategy to daily clinical practice is problematic [55]. For example, the increase of cardiac preload following fluid infusion may be inconsistent for each round of fluid infusion due to context-sensitive volume expansion. The absence of fluid responsiveness may simply be attributed to inadequate increases in cardiac preload following infusion. Accordingly, cardiac preload changes following every fluid bolus should be confirmed by flow-related dynamic parameters such as stroke volume variation (SVV) during mechanical ventilation which is closely related to cardiac preload [56].

Anastomotic leakage is a frequent complication of major abdominal surgery. Given that inadequate tissue perfusion can lead to anastomotic leakage, maintenance of tissue perfusion by adequate fluid administration may reduce the incidence of this complication. Kimberger et al. [57] demonstrated, in a pig model of colon anastomosis of 4-h duration, that goal-directed colloid administration (i.e., 3 ml kg^−1^ h^−1^ of Ringer’s acetate + bolus of 250 ml of 6 % HES 130/0.4) significantly increased microcirculatory blood flow in healthy and injured colon compared to goal-directed crystalloid administration (i.e., 3 ml kg^−1^ h^−1^ of Ringer’s acetate + bolus of 250 ml of Ringer’s acetate) or restrictive crystalloid fluid therapy (i.e., 3 ml kg^−1^ h^−1^ of Ringer’s acetate). As the cardiac index for goal-directed colloid administration was higher than those for other fluid therapies, it is difficult to separate the effects of systemic and regional hemodynamics on the improvement of microcirculatory blood flow in the colon for goal-directed colloid administration. Nevertheless, given that the splanchnic organs are at risk of hypoperfusion from hypovolemic insults, this finding confirms the scenario that improvements in microcirculatory blood flow in the gastrointestinal tract and colon contribute to the improvement of outcomes after major abdominal surgery by goal-directed fluid therapy using colloid solutions [52].

Norepinephrine infusion can reduce the total volume of fluid solution administered during major surgery. A randomized clinical study of radical cystectomy compared total fluid volume and the incidence of postoperative complications between a control group (6 ml kg^−1^ h^−1^ of balanced Ringer’s solution) and a group in which restrictive hydration (1–3 ml kg^−1^ h^−1^ of balanced Ringer’s solution) was combined with preemptive norepinephrine infusion to maintain MAP >60 mmHg (0.03–0.3 μg kg^−1^ min^−1^) [58]. Norepinephrine decreased the total volume of infused fluid solution by 60 % compared to the control (3.6 vs. 9.3 ml kg^−1^ h^−1^) and reduced the rates of gastrointestinal and cardiac complications compared to the control (26 vs. 85 %).

#### Trauma

Intravenous fluid administration is a first treatment for traumatic hemorrhage shock. However, as long as hemorrhage is not controlled, the full restoration of blood pressure by aggressive crystalloid volume loading may increase the risk of bleeding [59]. This is caused by dilution of coagulation factors leading to coagulopathy and increase of MAP that prevents clot formation [38].

According to the volume kinetic analysis, in normotensive adult male volunteers who had 900 ml of blood removed within 10–15 min, crystalloid infusion of 2700 ml over 30 min resulted in hypervolemia by 600 ml [59]. The crystalloid volume required to restore normovolemia was 1500 ml (i.e., 1.6 times the blood loss) much smaller compared to that previously recommended (i.e., three to four times the amount of blood loss) if fluid volume loading was started immediately after the hemorrhage. This finding is consistent with context-sensitive volume effect of infused fluid in that hypovolemia increases volume expanding effect of crystalloid solution [23]. Therefore, treatment of trauma with uncontrolled hemorrhage is “permissive resuscitation” that maintains MAP at 60 mmHg (i.e., avoid restoring MAP to normal levels) and avoid hypervolemia resulting from aggressive fluid volume loading [59].

Uncontrolled hemorrhage shock model in mice compared fluid requirements, blood loss, and intestinal microcirculation between fluid (i.e., 0.9 % saline) resuscitation with or without norepinephrine to target MAP at 50 and 60 mmHg [60]. The administration of norepinephrine significantly decreased fluid requirements by 60 % for MAP at 50 mmHg and 70 % for MAP at 60 mmHg compared to resuscitation only with fluid. Blood loss was comparable between two treatments for MAP at 50 mmHg but was halved by the use of norepinephrine compared to resuscitation only with fluid for MAP at 60 mmHg. The administration of norepinephrine with fluid volume loading preserved intestinal villi microcirculation for MAP at 50 and 60 mmHg. This beneficial effect of a combination of norepinephrine with fluid volume loading to reduce fluid requirements and blood loss while preserving microcirculation is attractive, but it remains to be confirmed in clinical trials observing outcomes from traumatic hemorrhage shock.

#### Sepsis

Sepsis occurs as a result of the systemic activation of inflammatory pathways by constituent parts of microorganisms. Early sepsis is characterized by a hyperdynamic vasodilatory state [61]. Fluid shift from the intravascular space to the interstitium due to capillary leakage causes hypovolemia and can decrease *P*_C_ despite vasodilation (Fig. 6c, red line). An important feature of microcirculation during sepsis is the decrease in capillary density and increase in heterogeneity of perfusion with non-perfused capillaries in close vicinity to well-perfused capillaries [61].

In the initial stages of sepsis, aggressive fluid administration is expected to restore microcirculation by reopening collapsed capillaries (Fig. 6c, blue line), given that low *P*_C_ increases the plasma volume expanding effect of the fluid solution. In severe sepsis and septic shock patients, both passive leg raising and volume expansion by normal saline or HES 130/0.4, increased vessel density and vessel perfusion and reduced microvascular heterogeneity in the sublingual microcirculation, within 24 h or their admission to the ICU [62]. Increases in cardiac output may have been responsible for the improved microcirculation. However, microcirculatory perfusion remained stable after cardiac output was further increased, suggesting that the relationship between improved microcirculation and increased cardiac output is not linear [62]. The use of vasopressor may preserve microcirculation via an increase of *P*_C_ due to increase of MAP (Fig. 6c, red broken line), but excessive use of vasopressor may decrease volume expanding effect of fluid solution and thus cause interstitial edema. Moreover, a previous multicenter observational study determined the influence of combined use of fluids and vasopressors on hospital mortality in septic shock patients [63]. In that study, retrospective evaluation using multivariable logistic regression showed that starting vasopressor in the initial hour after onset of septic shock without aggressive fluid administration may be detrimental given that pharmacologic vasoconstriction in the presence of hypovolemia could further impair tissue perfusion [63].

The benefit (e.g., reduced mortality) of early goal-directed therapy for septic shock that was originally proposed by Rivers et al. [64] has not been proven by recent systemic review with meta-analysis of five randomized clinical trials [65]. Given that early goal-directed therapy for septic shock patients requires aggressive fluid volume loading in the first 6 h based on MAP (≥65 mmHg), central venous pressure (≥8 mmHg), central venous oxygen saturation (≥70 %), and urine output (≥0.5 ml kg^−1^ h^−1^), resultant fluid overload causes increased use of fluid-related medical interventions such as diuresis and increased hospital mortality [66].

Once *P*_C_ is normalized by fluid administration, further fluid infusion may cause hypervolemia. The resultant increase in *P*_C_ enhances fluid leakage from the intravascular space to the interstitium due to degradation of the EG layer and ESL [1]. Therefore, aggressive fluid infusion should be restricted during the initial stages of sepsis (i.e., within 24 h). In one study, severe septic patients received 1000 ml of Ringer’s lactate solution or 400 ml of 4 % albumin solution either within 24 h (early) or more than 48 h (late) after a diagnosis of severe sepsis [67]. The administration of both fluids improved sublingual microvascular circulation in the early, but not late, stages of sepsis. These effects were independent of global hemodynamic effects and solution type.

#### Link between capillary hydrostatic pressure and fluid therapy in critical illness

Most frequent trigger for fluid volume loading in critical illness is hypotension. Indeed, low MAP may decrease *P*_A_, but low MAP does not necessarily imply low *P*_C_ because *P*_C_ is determined also by *P*_V_ and *R*_A_/*R*_V_. Given that *P*_C_ plays a key role for the volume expanding effect of fluid administration, strategy of fluid therapy differs by *P*_C_ (Table 1). However, monitoring *P*_C_ is difficult in a clinical setting, and therefore, we can only speculate whether *P*_C_ is low, normal, or high. A possible way to assess *P*_C_ is to observe hemodynamic responses to fluid volume loading (i.e., fluid challenge).

**Table 1:** Suggested fluid therapy to treat hypotension in critical illness on the theoretical basis of capillary hydrostatic pressure (*P*_C_)

Context	*P*_C_	Fluid volume loading	Vasopressor	Comments
Anesthesia	↑	(−)	(+)	
	↓	(+)	(+)	Low MAP can decrease *P*_C_ despite vasodilation.
Surgery				
Hemorrhage	↓	(+)	Use only when MAP is too low to preserve tissue perfusion.	
Long-duration	↓	Restrict crystalloid infusion (e.g., 3 ml kg^−1^ h^−1^). If hypovolemia is suspected, repeat crystalloid or colloid bolus (e.g., 4 ml kg^−1^ over 10–15 min).	Use if vasodilation due to inflammation is suspected.	
Trauma				
Uncontrolled bleeding	↓	(+)	Use only when MAP is too low to preserve tissue perfusion.	Avoid full restoration of MAP (≥60 mmHg) because of a risk of bleeding.
Sepsis				
Early	↓	(+)	Use only when MAP is too low to preserve tissue perfusion.	
After fluid volume loading	↑	(−)	(+)	

(+) recommended, (−) not recommended, MAP mean arterial pressure

The increase of MAP following fluid challenge may be a surrogate for detecting fluid responsiveness in clinical practice, but only 44 % of fluid responders (i.e., ≥10 % increase in cardiac output after 500 ml of saline or HES 130/0.4 over 30 min) in one study for septic shock patients showed an increase in MAP of more than 10 % from pre-infusion levels [68]. Fluid-induced reduction in arterial load in responders may explain the discrepancy between changes in MAP and cardiac output, given that intravascular volume expansion blunts baroreflex-mediated vasoconstriction in response to hypovolemia, reduces vascular tone via flow-mediated vascular relaxation, and recruits previously closed vessels [68].

ICU patients with a low sublingual microvascular flow index (MFI, <2.6) exhibited an increase in MFI after fluid challenge (500 ml of saline or 6 % HES 130/0.4 over 30 min), whereas those with a high sublingual MFI (≥2.6) showed no significant change [69]. However, fluid responsiveness (i.e., 10 % increase of stroke volume after fluid challenge) did not discriminate between MFI <2.6 or ≥2.6 at baseline. The discrepancy between MFI and fluid responsiveness suggests that the relationship between fluid responsiveness and restoration of microcirculation is complex and that fluid responsiveness does not imply the need for fluid therapy [69]. Given that low microvascular flow is associated with low *P*_C_ [14], the finding might support the scenario that low *P*_C_ increases volume expanding effect of fluid solutions.

Fluid challenge that monitors flow-related dynamic parameters following fluid bolus infusion is a useful tool for decision-making in fluid therapy [70]. For example, if the patients are hypovolemic in the condition of low *P*_C_ with vasoconstriction, they are expected to show fluid responsiveness (i.e., large increase of cardiac index or stroke volume) with large decrease of SVV after fluid challenge. In this context, fluid volume loading is effective to restore *P*_C_ via a large volume expansion effect and thus increases venous return and cardiac output.

Nevertheless, frequent fluid challenges result in excessive fluid administration causing tissue edema [71]. A recent study involving critically ill patients who were mechanically ventilated showed that change of SVV after mini-fluid challenge (100 ml colloid bolus during 1 min) accurately predicted fluid responsiveness that was defined as ≥15 % increase in cardiac index after the full (500 ml) fluid challenge [72]. In that study, SVV decreased by 3 % after fluid challenge and resulted in an increase of stroke volume index by 9.5 % for responders, whereas for non-responders, SVV remained unchanged. Given that SVV before fluid challenge was comparable between responders and non-responders (12 vs. 13 %), changes of SVV after fluid challenge rather than absolute values of SVV before fluid challenge may determine fluid responsiveness in those patients. Thus, mini-fluid challenge based on flow-related dynamic parameters may be a promising method to predict the effectiveness of fluid volume loading and possibly evaluate fluid status in critical illness given that it reduces a total amount of fluid required for frequent fluid challenges.

## Conclusions

Common to critically ill patients who undergo major surgery or suffer from trauma or sepsis are microcirculatory disturbances from capillary leakage due to inflammation. Although fluid administration is useful for restoring microcirculation by correcting hypovolemia, the volume expanding effects of the fluid solution is context-sensitive. A key element of these volume expanding effects may be capillary hydrostatic pressure, which depends on arterial pressure and vascular tone. The modulation of capillary hydrostatic pressure through the appropriate use of vasopressors might improve microcirculation and thus patient outcomes by increasing the volume effectiveness of fluid infusion and reducing the adverse effects of excessive fluid infusion. There remains a large gap between fluid pathophysiology based on the revised Starling equation and fluid therapy in critical illness because of difficulties in measuring capillary hydrostatic pressure in clinical settings. Nevertheless, clinicians should be aware of possible involvement of capillary hydrostatic pressure in a complexity of fluid therapy in critical illness.

## Abbreviations

COP: colloid osmotic pressure
EG: endothelial glycocalyx
ESL: endothelial surface layer
HES: hydroxyethyl starch
ICU: intensive care unit
MAP: mean arterial pressure
MFI: microvascular flow index
*P*_A_: arteriole hydrostatic pressure
*P*_C_: capillary hydrostatic pressure
*P*_V_: venule hydrostatic pressure
*R*_A_: hydraulic resistance in the arteriole
*R*_V_: hydraulic resistance in the venule
SVV: stroke volume variation
